# Balancing efficacy and hepatotoxicity: a comprehensive review of oral medications in psoriasis management

**DOI:** 10.1007/s00210-025-04334-1

**Published:** 2025-06-25

**Authors:** Alaa E. Elsisi, Sally El-Sayed Abu-Risha, Mahmoud Abdelrahman Alkabbani, Laila A. Ramadan, Samia Salem Sokar

**Affiliations:** 1 Department of Pharmacology & Toxicology, Faculty of Pharmacy, Tanta University, Tanta, Egypt; 2 Department of Pharmacology & Toxicology, Faculty of Pharmacy, Egyptian Russian University, Cairo, 11829 Egypt

**Keywords:** Acitretin, Apremilast, Cyclosporin, Hepatotoxicity, Methotrexate, Personalized medicine, Psoriasis

## Abstract

Psoriasis is a chronic, immune-mediated inflammatory disorder that significantly impacts patients’ quality of life. Oral systemic therapies, including methotrexate, cyclosporine, acitretin, and apremilast, remain integral to psoriasis management, particularly for patients with moderate-to-severe disease who cannot afford biologic therapy. While these treatments are effective, their long-term use is often limited by adverse effects, particularly hepatotoxicity. Methotrexate and acitretin are associated with liver toxicity, requiring regular monitoring, whereas cyclosporine presents a lower but notable risk. Apremilast, a phosphodiesterase 4 inhibitor, offers a safer hepatic profile but has lower efficacy than traditional systemic agents. Emerging therapies, such as TYK2 inhibitors, RORγt inhibitors, and novel PDE4 inhibitors, aim to improve treatment efficacy while minimizing adverse effects. Advances in understanding hepatotoxicity mechanisms have led to identifying predictive biomarkers and hepatoprotective strategies, including antioxidants and non-invasive imaging techniques. Personalized medicine approaches, including pharmacogenomics, are revolutionizing treatment selection by optimizing efficacy while minimizing toxicity risks. This comprehensive review examines oral psoriasis treatments’ efficacy and hepatotoxicity profiles, discusses novel therapeutic developments, and explores strategies for mitigating liver-related adverse effects. A balanced approach that integrates clinical monitoring, lifestyle modifications, and emerging precision medicine techniques is essential for optimizing long-term treatment outcomes. Future research should focus on refining predictive models for drug-induced liver injury and developing targeted therapies with improved efficacy and safety profiles.

**Graphical Abstract:**

Created in BioRender. Elgindy, A. (2025) https://BioRender.com/vcab653

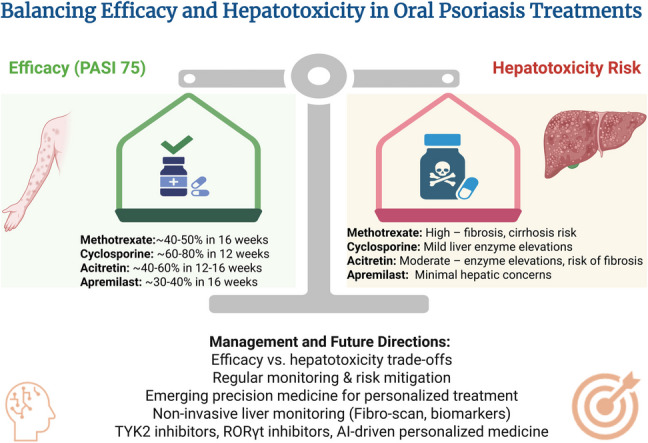

## Introduction

Psoriasis is a chronic, immune-mediated inflammatory skin disorder affecting approximately 2–3% of the global population. Recent estimates report over 40.8 million prevalent cases and 4.6 million new cases globally in 2019. It is characterized by excessive keratinocyte proliferation, resulting in the formation of scaly, erythematous plaques (Damiani et al. 2021; Vicic et al. 2021; Wang et al. 2024). The disease burden, while showing a global decline in age-standardized incidence rates between 1990 and 2019, remains significant and continues to exhibit substantial geographic and demographic disparities, particularly in individuals aged 40–64, the group with the highest incidence. By 2030, the global incidence of psoriasis is projected to rise to approximately 487.4 cases per 10,000 individuals, despite overall declining incidence rates, emphasizing the need for sustainable and targeted disease management strategies (Wang et al. 2024).

Psoriasis arises from a complex interplay of genetic predisposition, immune dysregulation, and environmental triggers (Ayala-Fontanez et al. 2016). Key inflammatory mediators implicated in its pathogenesis include interleukin (IL)−17, IL-23, and tumor necrosis factor-alpha (TNF-α), primarily released by activated dendritic cells and T-cells (Q. Li et al. 2024a, b; Mohammed et al. 2025; Ridha-Salman et al. 2025). The most prevalent form is plaque psoriasis, accounting for more than 80% of cases (Gupta et al. 2021), though other subtypes, such as pustular, erythrodermic, guttate, and inverse psoriasis, may require distinct therapeutic approaches (Kimmel & Lebwohl 2018; Salman et al. 2024). Psoriasis is also associated with systemic comorbidities, including psoriatic arthritis, cardiovascular disease, and metabolic syndrome, necessitating holistic treatment strategies (Bu et al. 2022).

Treatment options range from topical therapies and phototherapy to systemic agents and biologics (Ighani et al. 2019). For patients with moderate-to-severe disease, oral systemic therapies remain integral due to their cost-effectiveness and ease of administration (Lee & Kim 2023). Commonly used oral agents include methotrexate, cyclosporine, acitretin, and apremilast, each with distinct mechanisms of action and efficacy profiles (Menter et al. 2020).

Despite their therapeutic advantages, these oral medications are linked to a variety of possible side effects, with liver damage being a significant concern for drugs such as methotrexate and acitretin (Yelamos & Puig 2015; Balak et al. 2020). The risk of liver damage necessitates careful monitoring and management strategies to balance efficacy with safety. This delicate balance is critical to optimizing patient outcomes and minimizing the risk of long-term complications.

This review offers an integrative perspective by systematically comparing the efficacy and hepatotoxicity profiles of all approved oral psoriasis therapies while incorporating emerging treatments and pharmacogenomic considerations. This work emphasizes hepatotoxicity risk stratification, monitoring, and the potential role of predictive biomarkers and hepatoprotective strategies. It also proposes a structured framework to optimize treatment selection based on therapeutic benefit and hepatic safety, thereby addressing a clinically essential but underexplored dimension of long-term psoriasis care.

## Psoriasis overview

### Pathophysiology

**Fig. 1:**
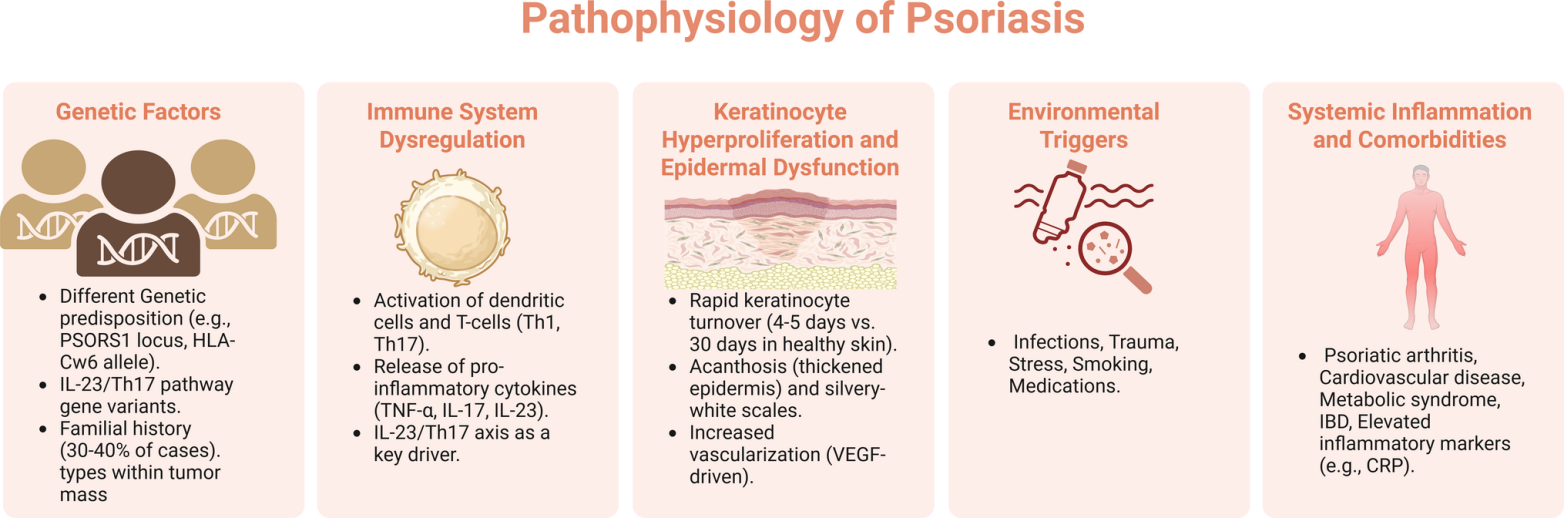
Pathophysiology of psoriasis; key processes involved in the development and progression of psoriasis

Psoriasis is a chronic, immune-mediated condition characterized by a complicated pathophysiology that includes genetic predisposition, environmental influences, and immune system dysregulation (Ayala-Fontanez et al. 2016). The condition is predominantly distinguished by the abnormal and excessive proliferation of keratinocytes in the epidermis, which results in the formation of thickened, scaly plaques on the skin (Pondeljak et al. 2024). Psoriasis fundamentally arises from the interaction between the innate and adaptive immune systems, particularly including T-cells, dendritic cells, and pro-inflammatory cytokines (Schon & Erpenbeck 2018) (Fig. 1). The underlying mechanisms of psoriasis can be categorized into several key processes, as detailed in the following paragraphs.

Created in BioRender. Elgindy, A. (2025) https://BioRender.com/zn90akz

#### Genetic factors

Psoriasis possesses a significant genetic component, with approximately 30–40% of affected individuals showing a familial history of the condition (Solmaz et al. 2020; Fortina & Caroppo 2022; Makene & Liu 2022). Specific gene mutations, notably those located in the PSORS1 locus on chromosome 6, linked to the human leukocyte antigen (HLA)-Cw6 allele, have been recognized as significant factors in psoriasis susceptibility (Dand et al. 2020; Trovato et al. 2022; Zalesak et al. 2024). Other genetic variants that influence immune-related genes, such as those associated with the IL-23/Th17 pathway, are also essential in the disease’s pathogenesis (Zalesak et al. 2024). While these genetic mutations cannot guarantee the development of psoriasis, they increase the probability of disease onset when combined with environmental triggers (Yan et al. 2021).

#### Immune system dysregulation

The immune system plays a central role in the pathogenesis of psoriasis, which is primarily considered a T-cell-mediated disorder involving both CD4 + helper and CD8 + cytotoxic T cells. In psoriatic lesions, hyperactivated dendritic cells release cytokines such as TNF-α, IL-12, and IL-23, which promote the differentiation of Th1 and Th17 cells (Chiricozzi et al. 2018; Hu et al. 2021). Th17 cells are vital, as they secrete IL-17 and IL-22, key drivers of keratinocyte hyperproliferation and the development of characteristic psoriatic plaques. As a result, the IL-23/Th17 axis has become a critical therapeutic target, with several biologic agents developed to inhibit this pathway (Deng et al. 2016; Bugaut & Aractingi 2021; Sharma et al. 2022).

#### Keratinocyte hyperproliferation and epidermal dysfunction

One of the hallmark features of psoriasis is the rapid turnover of keratinocytes in the epidermis. In healthy skin, keratinocytes take approximately a month to mature and move from the basal layer of the epidermis to the surface, where they are eventually shed. In psoriasis, however, this process is accelerated to just 4–5 days, leading to the accumulation of immature keratinocytes on the skin surface. This rapid proliferation is primarily driven by the pro-inflammatory cytokines secreted by Th1 and Th17 cells, particularly IL-17 and IL-22, which promote keratinocyte proliferation and inhibit their normal differentiation (Ortiz-Lopez et al. 2022).

The hyperproliferation of poorly differentiated keratinocytes leads to the thickening of the epidermis (acanthosis) and the formation of silvery-white scales on the surface of psoriatic plaques (Ortiz-Lopez et al. 2022; Singh et al. 2022). Additionally, psoriasis is associated with increased vascularization in the dermis, contributing to the erythematous appearance of the lesions (Jabeen et al. 2020; Negrutiu et al. 2024). The hypervascularization is driven by the release of vascular endothelial growth factor (VEGF) and other angiogenic factors (Luengas-Martinez et al. 2022; Chen et al. 2023; Parab & Doshi 2023).

#### Role of environmental triggers

While genetic and immunological factors are pivotal in the pathophysiology of psoriasis, environmental triggers are frequently essential to initiate or aggravate the condition (Yan et al. 2021). Common environmental triggers include infections (particularly streptococcal infections in guttate psoriasis), trauma to the skin (Koebner phenomenon), stress, smoking, and certain medications (e.g., lithium, beta-blockers). These triggers can activate the immune system and induce the release of inflammatory mediators, setting off the cascade that leads to psoriasis development or exacerbation (Garritsen et al. 2017; Ji & Liu 2019; Belloni Fortina & Caroppo 2022).

#### Comorbidities and systemic inflammation

Psoriasis is now increasingly recognized as a systemic inflammatory rather than an exclusively dermatological condition. The exact immune mechanisms that cause skin lesions can also contribute to inflammation in other organ systems, resulting in an elevated risk of comorbidities such as psoriatic arthritis, cardiovascular disease, metabolic syndrome, and inflammatory bowel disease (IBD) (Campanati et al. 2021). Chronic systemic inflammation in psoriasis patients is associated with heightened levels of inflammatory markers, including C-reactive protein (CRP), and an augmented risk of atherosclerosis and myocardial infarction (Visser et al. 2021; Zwain et al. 2021; Midtbo et al. 2022).

### Clinical presentation

Psoriasis is a heterogeneous condition presenting in several distinct clinical forms, each with specific morphological and anatomical features. The most common type is plaque psoriasis, or psoriasis vulgaris, which affects the majority of patients and manifests as well-demarcated, erythematous plaques with silvery-white scales, commonly on the scalp, elbows, and knees (Burden & Kirby 2016; Gupta et al. 2021). Lesions may be pruritic or painful and vary in size and distribution. The Auspitz sign (pinpoint bleeding upon scale removal) is a characteristic of plaque psoriasis (Kumar et al. 2024). Disease severity is typically assessed using indices such as the Psoriasis Area and Severity Index (PASI) (Wu et al. 2020; Leonardi et al. 2021; Papp et al. 2021).

Other forms include guttate psoriasis, which presents with numerous small, drop-like lesions and often follows streptococcal infection (Leung et al. 2023); inverse psoriasis, affecting intertriginous areas with smooth erythematous plaques and minimal scaling (Micali et al. 2019); and pustular psoriasis, characterized by sterile pustules on inflamed skin, which can be localized or generalized (Fujita et al. 2022). The generalized form of pustular psoriasis may cause systemic symptoms such as fever and can be life-threatening (Benjegerdes et al. 2016; Rivera-Diaz et al. 2023).

Erythrodermic psoriasis is a rare but severe variant involving widespread erythema and desquamation over most of the body surface, often requiring hospitalization due to risks such as fluid loss, electrolyte imbalance, and sepsis (González-Rivera et al. 2023; Potestio et al. 2023). Nail involvement occurs in approximately 40% of patients and up to 80% of those with psoriatic arthritis, presenting as pitting, onycholysis, subungual hyperkeratosis, and discoloration (Haneke 2017; Ji et al. 2021). Psoriatic arthritis, affecting up to 30% of psoriasis patients, can cause inflammatory joint pain, stiffness, swelling, and irreversible joint damage if untreated (Chimenti et al. 2019; Hioki et al. 2022). These diverse manifestations highlight the systemic nature of psoriasis and the need for tailored therapeutic strategies. A summary of the key clinical subtypes is provided in Table 1.

**Table 1:** Clinical forms of psoriasis

Psoriasis type	Key clinical features	Common locations	References
Plaque psoriasis (psoriasis vulgaris)	Raised, erythematous plaques with silvery-white scales; often symmetrical; may itch or burn	Elbows, knees, scalp, extensor surfaces	Burden and Kirby (2016); Gisondi et al. (2020b, c, a); Ranjitha (2020); Wu et al. (2020); Leonardi et al. (2021); Papp et al. (2021); Taliercio et al. (2021); Kumar et al. (2024)
Guttate psoriasis	Sudden eruption of small (< 1 cm), drop-like red lesions	Trunk and extremities	Sarac et al. (2016); Gananandan et al. (2020); Tambe et al. (2021); Leung et al. (2023)
Inverse psoriasis	Smooth, shiny, bright red plaques without scales	Intertriginous areas (groin, axillae, inframammary, genital)	Sarac et al. (2016); Micali et al. (2019); Gisondi et al. (2020b, c, a)
Pustular psoriasis	Sterile pustules on red, inflamed skin; systemic symptoms in severe cases	Localized: palms and soles; generalized: entire body	Benjegerdes et al. (2016); Fujita et al. (2022); Rivera-Diaz et al. (2023)
Erythrodermic psoriasis	Diffuse erythema, desquamation covering > 90% body surface; intense pruritus, pain, fever	Generalized skin	Shao et al. (2020); Reynolds et al. (2021); González-Rivera et al. (2023); Muhammed (2023); Potestio et al. (2023); Parks et al. (2024)
Nail psoriasis	Pitting, onycholysis, yellow–brown discoloration, subungual hyperkeratosis	Fingernails and toenails	Haneke (2017); Kara (2019); Ji et al. (2021); Stewart et al. (2021); Pala et al. (2023)
Psoriatic arthritis (PsA)	Joint pain, swelling, and stiffness may lead to erosion and deformity	Small joints of hands/feet, spine	Chimenti et al. (2019); Kishimoto et al. (2021); Hioki et al. (2022)

Understanding the diverse clinical manifestations of psoriasis is crucial for tailoring effective treatment strategies. The severity, distribution, and specific characteristics of lesions and associated comorbidities inform the selection of therapeutic modalities. For instance, while topical agents may suffice for localized mild disease, systemic therapies are often warranted for extensive or refractory cases (Rendon & Schakel 2019). Moreover, the presence of psoriatic arthritis or significant psychosocial impact may necessitate more aggressive interventions (Kim et al. 2017). Thus, a comprehensive assessment of clinical presentation is essential to guide optimal treatment decisions (Reid & Griffiths 2020).

### Current treatment landscape

An overview of the major therapeutic classes used in psoriasis management, including their mechanisms, clinical indications, representative agents, and key limitations, is summarized in Table 2. The therapeutic landscape for psoriasis is wide and varied, mirroring the intricate and diverse characteristics of the condition (Hedin et al. 2021). Treatment options vary from topical drugs for minor conditions to systemic therapies and biologics for moderate-to-severe disease conditions. The selection of treatment is contingent upon the type and severity of psoriasis, the extent of body surface involvement, patient comorbidities, and the response to prior therapies (Ighani et al. 2019; Kaushik & Lebwohl 2019a). The primary goals of psoriasis therapy are to achieve disease control and enhance the patient’s quality of life, focusing on inflammation reduction, symptom alleviation, and the prevention of comorbidities related to the condition (Kaushik & Lebwohl 2019a; Svoboda et al. 2020; Belinchon Romero et al. 2021).

**Table 2:** Summary of current psoriasis treatment modalities: mechanisms, clinical use, and limitations

Class/agent	Mechanism of action	Indications	Common adverse effects/limitations	References
Topical therapies				
Corticosteroids	Anti-inflammatory, immunosuppressive; ↓ cytokines and cell proliferation	Mild-to-moderate plaque psoriasis	Skin atrophy (especially with prolonged use), tachyphylaxis	Torsekar and Gautam (2017); Kleyn et al. (2019); Bakshi et al. (2020); Lo et al. (2024)
Vitamin D analogs (e.g., calcipotriene)	Regulate keratinocyte differentiation and proliferation	Often combined with steroids, long-term control	Mild irritation, less effective as monotherapy	Bark et al. (2022); Taylor et al. (2023); Gisondi et al. (2024)
Calcineurin inhibitors (e.g., tacrolimus)	Inhibit T-cell activation by blocking calcineurin	Facial, genital, and intertriginous psoriasis	Local burning, cost, and limited evidence base	Dattola et al. (2018); Luk et al. (2021)
Retinoids (e.g., tazarotene)	Normalize epidermal differentiation, anti-inflammatory	Mild-moderate psoriasis; often adjunctive	Irritation, teratogenicity	Han et al. (2020); Lebwohl et al. (2021)
Phototherapy				
NB-UVB	Induces T-cell apoptosis, reduces keratinocyte proliferation	Moderate disease unresponsive to topicals	Skin aging, ↑ long-term cancer risk	Lin et al. (2019); Ye et al. (2020); Borgia et al. (2022); Sarsik et al. (2025)
Systemic oral therapies				
Methotrexate	DHFR inhibition → ↓ DNA synthesis; ↓ inflammation	Moderate-to-severe psoriasis	Hepatotoxicity, cytopenias require monitoring	Nedelcu et al. (2019); Mazaud and Fardet (2021); El-Dessouki et al. (2025)
Acitretin	Retinoid: modulates keratinocyte differentiation	Moderate-to-severe, or pustular psoriasis; not immunosuppressive	Teratogenicity, mucocutaneous effects, hepatotoxicity	Chen et al. (2018); Jiang et al. (2019); Sarkar and Meena (2023); Younis et al. (2023)
Cyclosporine	Calcineurin inhibitor; ↓ T-cell activation	Moderate-to-severe psoriasis	Nephrotoxicity, hypertension, and immunosuppression	Rafael-Vidal et al. (2021); Nouri et al. (2022); Singh et al. (2024)
Apremilast	PDE4 inhibition → ↓ TNF-α, IL-17, IL-23	Moderate-to-severe psoriasis	GI upset (nausea, diarrhea), weight loss	Li et al. (2018); Vujic et al. (2018); Tsentemeidou et al. (2022)
Biologic therapies				
TNF-α inhibitors (e.g., etanercept, infliximab, adalimumab)	Neutralize TNF-α; ↓ inflammation and keratinocyte activation	Moderate-to-severe plaque psoriasis	Infection risk, latent TB reactivation	Campanati et al. (2019); Furiati et al. (2019); Gisondi et al. (2020b, c, a); Nast et al. (2021)
IL-17 inhibitors (e.g., secukinumab, ixekizumab)	Block IL-17A/F; ↓ Th17-mediated inflammation	Moderate-to-severe psoriasis; high efficacy	Candida infections, IBD risk	Brembilla et al. (2018); Tiburca et al. (2022)
IL-23 inhibitors (e.g., guselkumab, tildrakizumab)	Target p19 subunit of IL-23 → ↓ Th17 differentiation	Long-term control in moderate-to-severe cases	Injection site reactions, rhinitis	Fragoulis and Siebert (2022); Borriello et al. (2024)
Emerging therapies				
JAK inhibitors (e.g., tofacitinib, deucravacitinib)	Inhibit JAK-STAT signaling → ↓ cytokine signaling	Moderate-to-severe psoriasis	Potential risks of infection, malignancies, and thrombosis	Krueger et al. (2022); Furtunescu et al. (2024); Truong et al. (2024)

Topical treatments are the primary treatment for patients with mild-to-moderate psoriasis. This encompasses corticosteroids, vitamin D analogs (e.g., calcipotriene), calcineurin inhibitors (such as tacrolimus for sensitive areas), and retinoids (such as tazarotene) (Torsekar & Gautam 2017; Kleyn et al. 2019). Corticosteroids are the most frequently prescribed topical medications owing to their anti-inflammatory and immunosuppressive properties, which alleviate erythema, scaling, and plaque thickness (Kang et al. 2021). Topical corticosteroids are the most frequently prescribed medications due to their immunosuppressive and anti-inflammatory properties, which reduce plaque thickness, scaling, and erythema. However, long-term use of topical corticosteroids can result in adverse effects, including skin atrophy, particularly in sensitive areas. Consequently, their use is frequently limited in duration or combined with other agents (Kleyn et al. 2019; Niculet et al. 2020; Kang et al. 2021). Vitamin D analogs, commonly used with combined corticosteroids, assist in normalizing keratinocyte proliferation and are generally well-tolerated for longer-term treatment (Segaert et al. 2017). The use of calcineurin inhibitors is recommended for sensitive areas, including the face, genitalia, and intertriginous regions, due to their reduced potential for causing skin atrophy (Amiri et al. 2023).

Phototherapy is an efficacious treatment approach for patients with extensive psoriasis who do not sufficiently respond to topical medications alone. Narrowband ultraviolet B (NB-UVB) phototherapy is preferable, as it efficiently diminishes psoriatic plaques with fewer adverse effects than traditional broadband UVB phototherapy. Phototherapy is frequently administered two to three times per week under clinical supervision and may be coupled with topical medications to augment efficacy. Although phototherapy is often safe, extended exposure may elevate the risk of skin cancer; hence, cumulative UV exposure is monitored in patients necessitating continuous treatment (Krenitsky et al. 2020; Torres et al. 2021; Mahajan et al. 2022; Sreya et al. 2023).

Regarding moderate therapies, systemic treatments are often advised for psoriasis or those refractory to topical and phototherapy treatments. Among these are oral medications that include acitretin, cyclosporine, methotrexate, and apremilast (Ighani et al. 2019). Methotrexate, a folate antagonist, has been utilized in the treatment of psoriasis for decades, demonstrating efficacy in diminishing inflammation and inhibiting keratinocyte hyperproliferation (Elango et al. 2022). It is particularly beneficial for patients with both skin and joint involvement, as it is also used in the management of psoriatic arthritis (Elmamoun & Chandran 2018). Nevertheless, methotrexate has considerable adverse effects, particularly hepatotoxicity, which necessitates regular monitoring of liver function and adaptations to the dosage based on patient tolerance (Barnhill et al. 2020; van de Meeberg et al. 2023).

Apremilast, a newer oral drug, is a phosphodiesterase 4 (PDE4) inhibitor that regulates inflammation by reducing the production of pro-inflammatory cytokines (Perez-Aso et al. 2015). It has effectively alleviated psoriatic symptoms, especially in individuals with moderate-to-severe conditions. Apremilast is often well-tolerated and possesses a good safety profile relative to conventional systemic treatments; nonetheless, it is linked to gastrointestinal adverse effects, notably nausea and diarrhea, which may restrict its application in some individuals (Papp et al. 2015; Perez-Aso et al. 2015; Aljefri et al. 2022; Merola et al. 2024).

Biologic therapies have revolutionized the management of moderate-to-severe psoriasis, particularly for individuals who do not respond to conventional systemic treatments. These drugs have been designed to target particular elements of the immune system implicated in psoriasis etiology, delivering very effective and prolonged symptom alleviation (Wu et al. 2024). TNF-α inhibitors, including etanercept, infliximab, and adalimumab, represent the first class of biologics employed in psoriasis treatment, exhibiting substantial success in diminishing psoriatic plaques and enhancing quality of life (Megna et al. 2022; Nikam et al. 2023; Ferrara et al. 2024). However, introducing newer biologics targeting the IL-17 and IL-23 cytokine pathways has provided even more effective and targeted options. In clinical trials, IL-17 inhibitors (e.g., secukinumab and ixekizumab) and IL-23 inhibitors (e.g., guselkumab and tildrakizumab) have been associated with high rates of complete or near-complete cutaneous clearance. These inhibitors specifically modulate the inflammatory pathways central to psoriasis. Ongoing dosing is typically required for biologics, which are usually administered via injection, although the frequency of administration differs by agent (Gooderham et al. 2018; Potestio et al. 2024).

The severity of the disease determines the individualization of treatment for each patient, the response to previous therapies, and the potential for adverse effects. Topical medications are usually effective for managing mild-to-moderate cases; individuals with moderate-to-severe psoriasis typically need systemic medication and phototherapy to attain satisfactory disease control (Ighani et al. 2019; Arora et al. 2021). For severe cases, biologics and traditional systemic agents like methotrexate, retinoids, and cyclosporine are more common choices (Nakamura & Koo 2020; Arora et al. 2021). Combination therapies are also employed to improve efficacy and mitigate adverse effects, for example, combining methotrexate with a biologic to reduce immunogenicity or combining phototherapy with topical treatments to achieve greater plaque clearance (Arora et al. 2021; Sreya et al. 2023).

In recent years, clinical trials have demonstrated the potential of emerging therapies, which focus on targeting specific inflammatory pathways implicated in psoriasis, such as the IL-23/Th17 axis. Small-molecule inhibitors targeting Janus kinase (JAK) pathways are also being investigated, with the potential to broaden the variety of treatment options available to psoriasis patients (Papp et al. 2016; Thakur & Mahajan 2022).

## Oral medications for psoriasis

**Fig. 2:**
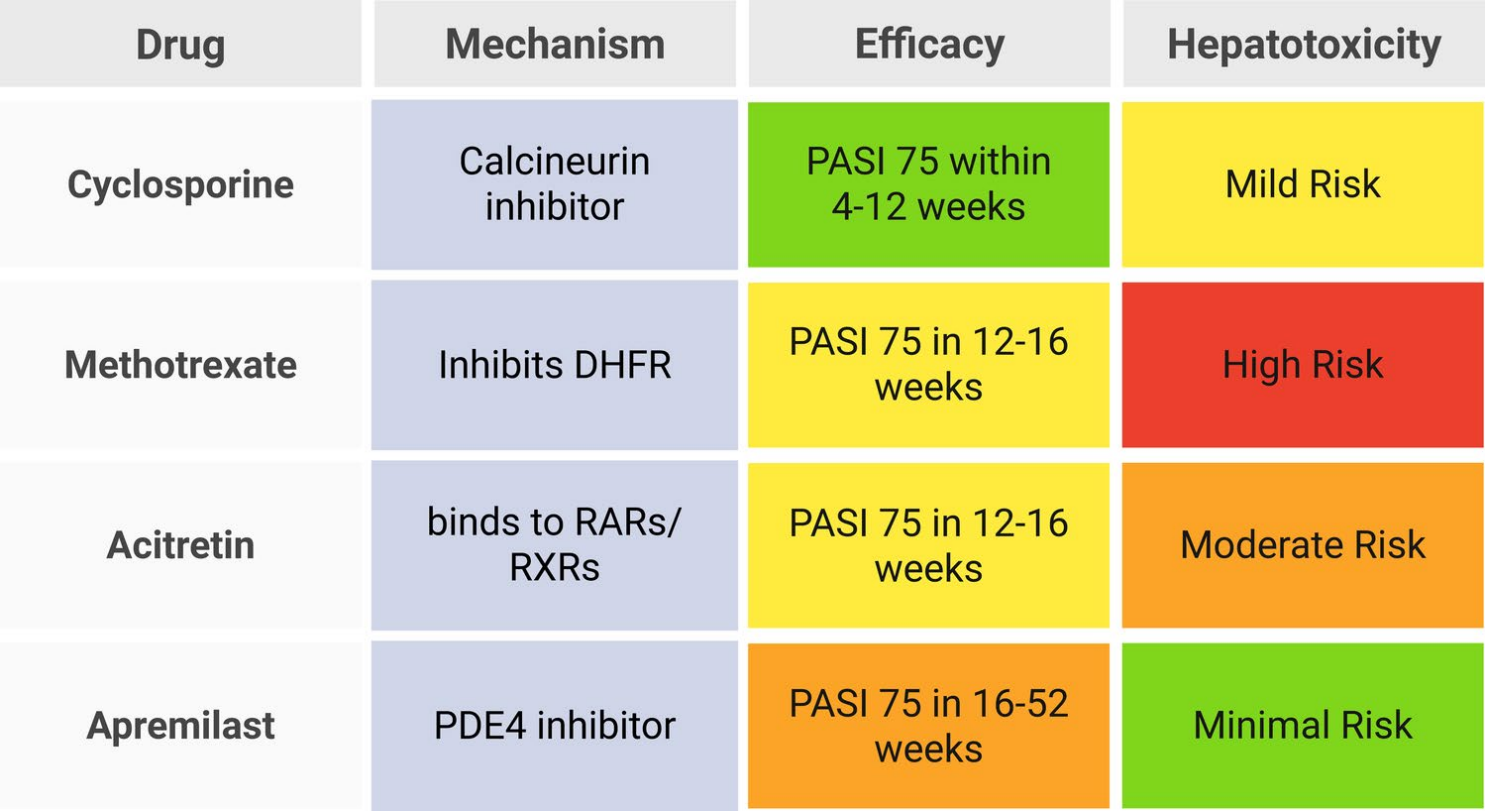
Overview of oral medications for psoriasis: mechanisms, efficacy, and hepatotoxicity risks. Created in BioRender. Elgindy, A. (2025) https://BioRender.com/2u0xp2j

Figure 2 summarizes the four main oral medications (methotrexate, cyclosporine, acitretin, apremilast) and their key attributes.

### Methotrexate

Methotrexate is one of the most established and widely used systemic treatments for moderate-to-severe psoriasis (Carrascosa et al. 2016). Originally developed as a chemotherapy agent, methotrexate was later found to be effective in managing autoimmune diseases, including psoriasis and rheumatoid arthritis (Kozminski et al. 2020). Its widespread use is attributed to its efficacy, relatively low cost, and extensive clinical experience. However, methotrexate therapy requires careful monitoring due to its potential for adverse effects, particularly hepatotoxicity (Malaviya 2016; Boh et al. 2021; Turner 2023).

#### Mechanism of action

Methotrexate functions predominantly as an immunosuppressant and antimetabolite. It inhibits dihydrofolate reductase (DHFR), an enzyme crucial for converting dihydrofolate to tetrahydrofolate, which is essential for synthesizing DNA and RNA. By blocking this pathway, methotrexate inhibits the proliferation of rapidly dividing cells, such as activated T-cells in the immune system and keratinocytes in the skin (Kim et al. 2016; Friedman & Cronstein 2019; Nedelcu et al. 2019).

Methotrexate inhibits the excessive proliferation of keratinocytes in psoriasis, thereby reducing the formation of plaques. It also exhibits potent anti-inflammatory effects by inhibiting the production of pro-inflammatory cytokines, including TNF-α and IL-6, and increasing the release of anti-inflammatory adenosine. Methotrexate is an effective treatment for psoriasis-associated comorbidities, such as psoriatic arthritis due to its dual efficacy in modulating the immune response and reducing keratinocyte proliferation (Elango et al. 2022).

#### Efficacy

Methotrexate is considered a cornerstone therapy for moderate-to-severe psoriasis owing to its cost-effectiveness, efficacy, and extensive clinical history. Its immunosuppressive mechanism specifically targets rapidly proliferating keratinocytes and attenuates the inflammatory cascade by diminishing pro-inflammatory cytokines, including TNF-α and IL-17, which are pivotal to the pathogenesis of psoriasis (Carrascosa et al. 2016; Rajitha et al. 2017; Friedman & Cronstein 2019; Elango et al. 2022). It has been shown to attain PASI 75 in a substantial proportion of patients within 12 to 16 weeks in clinical trials, resulting in significant skin clearance and quality-of-life improvements (Carrascosa et al. 2016; Elmamoun & Chandran 2018).

Methotrexate’s versatility is particularly evident in its efficacy for both cutaneous and joint manifestations of psoriatic disease. Studies have shown its ability to improve symptoms of psoriatic arthritis, alleviating joint pain, swelling, and stiffness, thus making it a dual-action treatment for patients with systemic involvement (Wilsdon et al. 2019; Boh et al. 2021). Furthermore, real-world observational studies highlight its effectiveness in patients with refractory psoriasis or those who cannot afford biologics, where methotrexate remains a first-line systemic therapy (Nedelcu et al. 2019; Zhang et al. 2024). Despite its advantages, efficacy is dose-dependent, and higher doses are often limited by the risk of adverse effects, particularly hepatotoxicity (Yelamos & Puig 2015).

Methotrexate has been shown to improve outcomes when used in conjunction with other therapies. For example, the incorporation of phototherapy or biologics into methotrexate regimens frequently enhances plaque clearance rates and decreases the likelihood of developing resistance (Heath et al. 2019; Ara et al. 2020; Xie et al. 2021; Chao et al. 2024). Long-term efficacy studies indicate that many patients can achieve and maintain reasonable disease control with a continuous treatment methodology, especially when combined with folic acid supplementation to mitigate side effects. Overall, methotrexate remains an indispensable option in psoriasis management, particularly for those seeking a balance between efficacy and cost (Murage et al. 2018; Friedman & Cronstein 2019; Mazhar et al. 2023).

#### Hepatotoxicity

Despite the efficacy of methotrexate in a variety of diseases, such as cancer and autoimmune diseases, its clinical utility is frequently overshadowed by its hepatotoxic potential. Methotrexate-induced liver toxicity is primarily caused by apoptosis, inflammation, and oxidative stress. Oxidative stress is exacerbated by methotrexate, which induces lipid peroxidation and depletes antioxidants like glutathione (GSH). This results in the accumulation of reactive oxygen species (ROS) and mitochondrial dysfunction. Moreover, methotrexate exacerbates hepatocyte toxicity by impairing endoplasmic reticulum (ER) function (Dogra et al. 2021; Ezhilarasan 2021; Schmidt et al. 2022; Sweilam et al. 2024).

The buildup of methotrexate-polyglutamate, a metabolite of methotrexate, promotes its hepatotoxic effects. Methotrexate-polyglutamate stimulates pro-inflammatory cytokines, such as TNF-α, IL-6, and nuclear factor-κ B (NF-κB), initiating inflammatory reactions that damage hepatocytes. It also diminishes intracellular folate levels, impairing nucleic acid synthesis and inducing hepatocyte apoptosis. This process stimulates hepatic stellate cells (HSCs), resulting in extracellular matrix deposition and fibrosis (Ezhilarasan 2021; Al-Khawalde et al. 2022; Schmidt et al. 2022; Alkabbani et al. 2024).

Several investigations have identified potential therapeutic options to reduce methotrexate-induced hepatotoxicity. Melatonin, naringin, ferulic acid, glabridin, curcumin, and vitamin C are examples of agents that have been demonstrated to restore redox balance, minimize oxidative damage, and suppress inflammation. Pretreatment with these agents successfully reduces serum transaminase levels and histopathological liver alterations. Melatonin, for instance, decreases lipid peroxidation, boosts antioxidant levels, and inhibits caspase-3 activity, all of which maintain hepatocyte integrity (Elsawy et al. 2020; Roghani et al. 2020; Dogra et al. 2021; Hasan Khudhair et al. 2022; Abdallah et al. 2024). Similarly, punicalagin promotes nuclear factor erythroid 2-related factor 2 (Nrf2) signaling, which boosts antioxidant defenses while decreasing apoptosis and inflammation (Al-Khawalde et al. 2022).

Patients with underlying disorders, such as non-alcoholic fatty liver disease (NAFLD) and IBD, are more susceptible to methotrexate-induced hepatotoxicity. Methotrexate exacerbates NAFLD by inhibiting antioxidant activity, boosting lipid buildup, and generating mitochondrial dysfunction, which leads to increased hepatocyte apoptosis and inflammation. This emphasizes the importance of complete hepatic evaluation and careful methotrexate usage in individuals with metabolic liver disorders (Ezhilarasan 2021; Wang et al. 2021; Schmidt et al. 2022; Berkemeyer et al. 2024). Furthermore, methotrexate’s interaction with liver cells goes beyond its pharmacological activity to include modification of gut microbiota and systemic immunological responses. Methotrexate therapy has been demonstrated in animal models to modify the intestinal microbiome, which may result in increased gut permeability and the translocation of bacterial products into the systemic circulation (Xia et al. 2022).

**Fig. 3:**
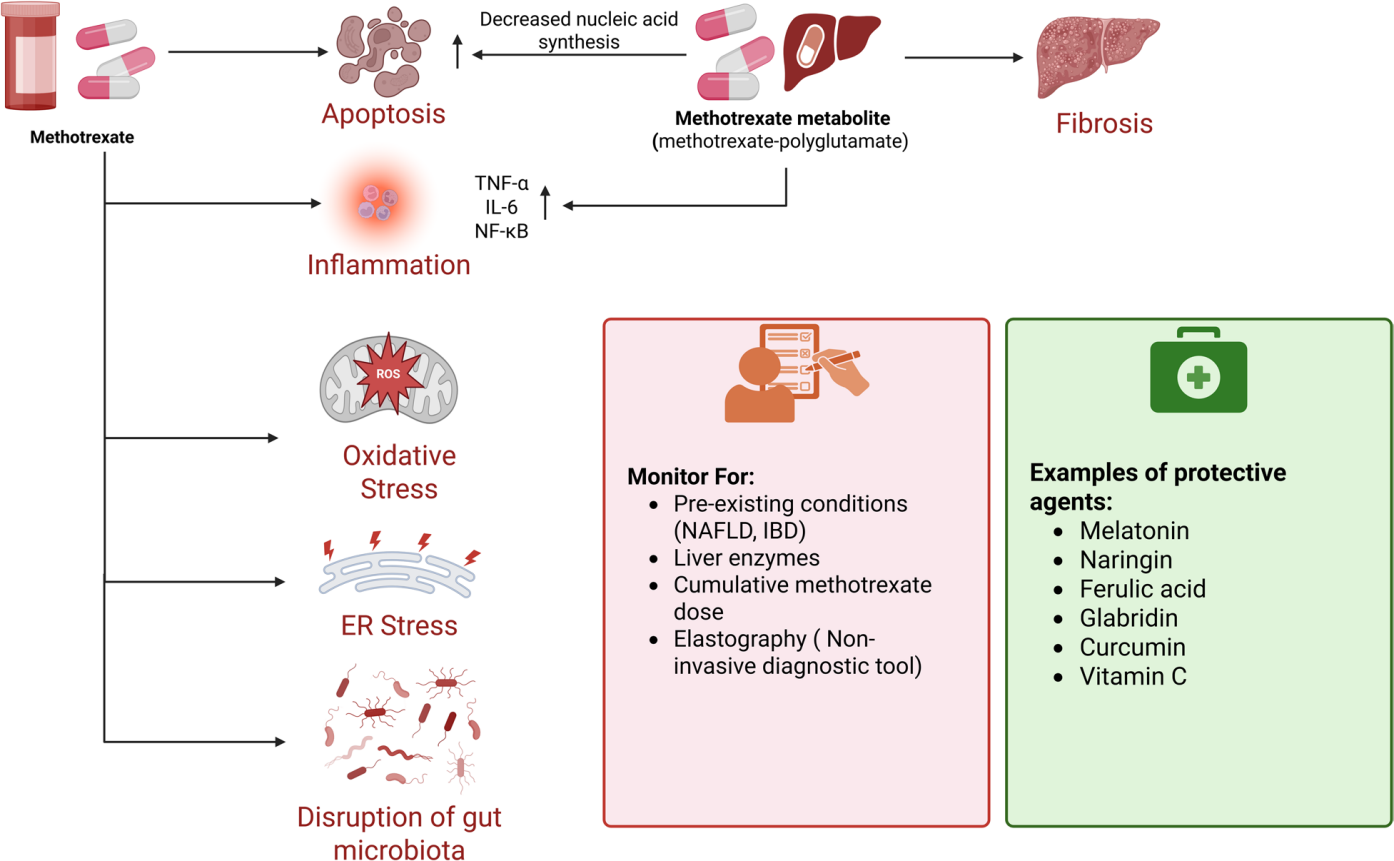
Key mechanisms of methotrexate-induced hepatotoxicity, key monitoring strategies, and examples of protective agents. Created in BioRender. Elgindy, A. (2025) https://BioRender.com/3yqir0q

Contrary to previous assumptions, emerging research shows that the cumulative dosage of methotrexate may not be the only factor influencing chronic hepatotoxicity. Transient increases in liver enzymes are common and frequently resolve without causing substantial fibrosis in persons with healthy livers. Elastography, a non-invasive diagnostic tool, is increasingly suggested for monitoring liver function in patients following long-term methotrexate therapy (Azzam et al. 2021; Di Martino 2023; Di Martino et al. 2023). Figure 3 summarizes the key mechanisms of methotrexate-induced hepatotoxicity, key monitoring strategies, and examples of protective agents.

### Cyclosporine

Cyclosporine is a systemic immunosuppressant extensively employed in treating moderate-to-severe psoriasis. It is particularly effective in cases requiring rapid disease control, such as acute exacerbation or severe disease flares. Despite its efficacy, cyclosporine is frequently restricted to short-term therapy due to its potential for significant adverse effects, such as hepatotoxicity, hypertension, and nephrotoxicity (Singh & Argáez 2018; Pandey et al. 2022).

#### Mechanism of action

Cyclosporine is a calcineurin inhibitor that suppresses the immune system by inhibiting T-cell activation. It forms a complex with cyclophilin, a cytoplasmic protein present in T-cells, that inhibits the activity of calcineurin, a calcium/calmodulin-dependent phosphatase. Calcineurin is a transcription factor that is essential for the activation of nuclear factor of activated T-cells (NFAT), which is responsible for the production of pro-inflammatory cytokines, including IL-1β, IL-2, IL-6, IL-4, and interferon (IFN)-γ (Pandey et al. 2022; Singh et al. 2024; Yang et al. 2024).

By blocking these cytokines, cyclosporine reduces the proliferation and activation of T-cells, which are central to the inflammatory cascade in psoriasis. The inhibition of T-cell activity ultimately decreases keratinocyte hyperproliferation and inflammation, improving psoriatic plaques. This targeted immunosuppressive mechanism accounts for the drug’s rapid onset of action and high efficacy in controlling severe psoriasis symptoms (Singh & Argáez 2018; Boh et al. 2021).

#### Efficacy

Cyclosporine is highly effective in treating moderate-to-severe psoriasis, particularly when rapid disease control is required. Clinical trials have shown that cyclosporine achieves significant skin clearance, often within a few weeks of starting treatment. Patients typically experience an improvement in PASI scores as early as 4 weeks, with sustained efficacy throughout the treatment period (Singh & Argáez 2018).

Studies have shown that cyclosporine can achieve PASI 75 (a 75% reduction in PASI scores) in 60–80% of patients within 12–16 weeks, particularly when combined with low-dose methotrexate. This makes it ideal for patients who have severe flares, erythrodermic psoriasis, or are unresponsive to other treatments. Cyclosporine’s rapid onset of action makes it an effective bridge therapy for patients transitioning to slower-acting systemic treatments, in transition strategies between different regimens, and during exacerbations (Singh & Argáez 2018; Balak et al. 2020; Singh & Singnarpi 2021; Dogra et al. 2024).

However, the long-term use of cyclosporine is generally avoided due to cumulative toxicity risks, and treatment is often limited to 12–24 months. The drug is most effective in intermittent, short-term courses, providing symptom control while minimizing adverse effects (Balak et al. 2020; Rajagopalan et al. 2022; Akarsu 2023).

#### Hepatotoxicity

**Fig. 4:**
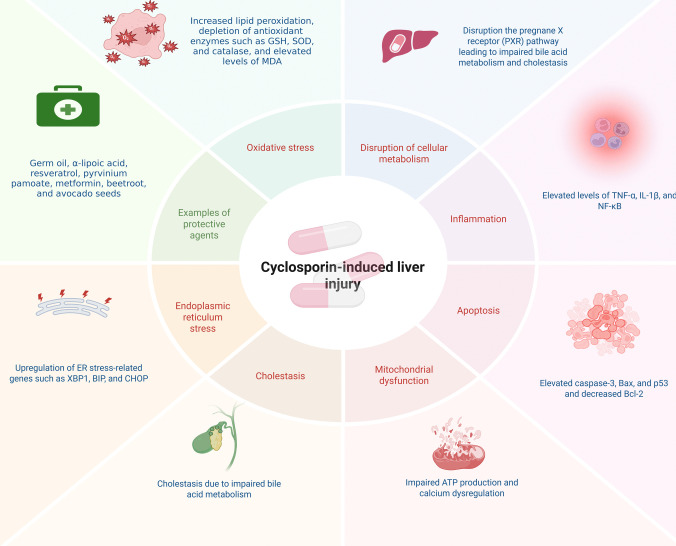
Schematic representation of the mechanisms underlying cyclosporin-induced hepatotoxicity and corresponding protective strategies. Created in BioRender. Elgindy, A. (2025) https://BioRender.com/jarg0cr

Despite its anti-psoriatic and broad-spectrum immunosuppressive efficacy, cyclosporin’s clinical use is frequently limited by significant side effects, including hepatotoxicity. Cyclosporin-induced liver injury is caused by complex mechanisms that include oxidative stress, inflammation, apoptosis, and disruption of cellular metabolism. Several studies have investigated these mechanisms and potential protective strategies to reduce cyclosporin-induced liver damage (Fig. 4) (Safaa Ahmed Faheem et al. 2022a, b).

One of the primary mechanisms of cyclosporin-induced hepatotoxicity is the production of oxidative stress. Cyclosporin administration has been shown to increase ROS production, causing lipid peroxidation, depletion of antioxidant enzymes such as GSH, superoxide dismutase (SOD), and catalase, and elevated levels of malondialdehyde (MDA) (Akool el 2015; Korolczuk et al. 2016; Bingul et al. 2021; S. A. Faheem et al. 2022a, b). This oxidative imbalance results in cellular damage, as evidenced by increased liver enzymes such as alanine transaminase (ALT) and aspartate transaminase (AST) (; Korolczuk et al. 2016; Albalawi et al. 2019). Furthermore, cyclosporin-induced oxidative stress has been associated with mitochondrial dysfunction, which is characterized by impaired adenosine triphosphate (ATP) production and calcium dysregulation, thereby exacerbating liver injury (Korolczuk et al. 2016; Nsengimana et al. 2022).

Cyclosporin treatment has been demonstrated to upregulate pro-inflammatory cytokines TNF-α, IL-1β, and NF-κB, contributing to hepatic inflammation and tissue damage. Inflammation also plays a critical role in cyclosporin-induced hepatotoxicity (El-Sherbeeny & Nader 2016; El-Mancy et al. 2022; S. A. Faheem et al. 2022a, b). The activation of these inflammatory pathways is often accompanied by increased expression of inducible nitric oxide synthase (iNOS) and cyclooxygenase-2 (COX-2), further promoting liver injury (Vangaveti et al. 2021; El-Mancy et al. 2022). Moreover, cyclosporin has been found to disrupt the pregnane X receptor (PXR) pathway, which regulates drug-metabolizing enzymes and transporters, leading to impaired bile acid metabolism and cholestasis (Shang et al. 2024).

Apoptosis is an additional significant mechanism in the development of hepatotoxicity induced by cyclosporin. Pro-apoptotic markers, including caspase-3, Bax, and p53, are upregulated by cyclosporin treatment, while anti-apoptotic proteins, such as Bcl-2, have been decreased (El-Sherbeeny & Nader 2016; Albalawi et al. 2019; El-Magd et al. 2022; El-Mancy et al. 2022; S. A. Faheem et al. 2022a, b). This imbalance triggers programmed cell death in hepatocytes, contributing to liver dysfunction. ER stress has also been implicated in cyclosporin-induced apoptosis, with studies showing upregulation of ER stress-related genes such as *XBP1*, *BIP*, and *CHOP* (El-Magd et al. 2022). These findings emphasize the complex relationship between apoptosis, inflammation, and oxidative stress in cyclosporin-induced liver injury.

Several studies have investigated potential therapeutic interventions to mitigate cyclosporin-induced hepatotoxicity. Antioxidants such as wheat germ oil, α-lipoic acid, and resveratrol have demonstrated protective effects by reducing oxidative stress and inflammation (Akool el 2015; Bingul et al. 2021; El-Mancy et al. 2022). Similarly, compounds like pyrvinium pamoate and metformin have shown promise in modulating signaling pathways such as Wnt/β-catenin and peroxisome proliferator-activated receptor (PPAR)-γ, thereby alleviating liver damage (Vangaveti et al. 2021; S. A. Faheem et al. 2022a, b). Additionally, natural extracts like beetroot and avocado seeds have been found to ameliorate cyclosporin-induced hepatotoxicity by restoring redox balance and reducing apoptosis (Albalawi et al. 2019; El-Magd et al. 2022).

### Acitretin

Acitretin is a second-generation derivative of vitamin A and an oral retinoid that is predominantly employed in the treatment of moderate-to-severe psoriasis (Heath et al. 2018). In the 1980 s, it was approved for clinical use (Abuarij et al. 2024). It is particularly effective in pustular and erythrodermic psoriasis, but it is also used as an adjunct therapy in plaque psoriasis (Singh et al. 2016; Subedi et al. 2018). Its ability to normalize keratinocyte differentiation and regulate inflammation makes it a valuable systemic option, particularly for patients unable to afford biologic therapies (Natsis et al. 2020; Molinelli et al. 2024).

#### Mechanism of action

Acitretin exerts its effects by binding to retinoic acid receptors (RARs) and retinoid X receptors (RXRs), leading to gene transcription changes that mediate its anti-proliferative and anti-inflammatory properties. By normalizing keratinocyte differentiation and proliferation, acitretin contributes to the resolution of hyperkeratosis and parakeratosis in psoriatic lesions. Additionally, it promotes epidermal differentiation, inhibits sebocyte proliferation, exhibits antineoplastic effects, and aids wound healing by modulating apoptosis and enhancing the synthesis of mucopolysaccharides and collagen (Lin et al. 2016; Heath et al. 2019).

Acitretin furthermore demonstrates immunomodulatory properties by diminishing the secretion of pro-inflammatory cytokines, such as IL-6 and TNF-α, which are pivotal in the pathogenesis of psoriasis (Heath et al. 2018; Agnihotri et al. 2023). Moreover, it affects neutrophil and T-cell function, attenuating inflammatory pathways linked to psoriatic plaques (Zhou et al. 2017; Agnihotri et al. 2023). The combination of acitretin with other treatments, such as matrine, has been explored to enhance anti-inflammatory effects and improve outcomes in psoriasis models (Jiang et al. 2019).

#### Efficacy

Acitretin has shown significant effectiveness in several psoriasis subtypes, especially in pustular, erythrodermic, and nail psoriasis (Singh et al. 2016; Subedi et al. 2018; Yu et al. 2023). A multicenter retrospective study showed that acitretin treatment led to substantial clinical improvement, attaining PASI 75 in individuals with moderate-to-severe psoriasis (Di Lernia et al. 2016).

Combination therapies, including acitretin and NB-UVB phototherapy, have shown improved therapeutic results while minimizing cumulative phototherapy doses. This combined strategy is very efficacious in attaining rapid disease management (Arora et al. 2021; Sreya et al. 2023). For nail psoriasis, acitretin monotherapy significantly reduces NAPSI scores over time (Pasch 2016; Hwang & Lipner 2023).

Despite its efficacy, acitretin is often limited by its side effect profile, including mucocutaneous dryness and hyperlipidemia. These adverse effects are dose-dependent, emphasizing the need for individualized dosing regimens. The drug’s teratogenic potential requires strict contraception for women of childbearing age, as highlighted in global treatment guidelines (Simin & Nagesh 2020; Sadowska et al. 2022). Additionally, hepatotoxicity is a notable concern, particularly in patients with risk factors such as alcohol consumption, obesity, and diabetes, which can exacerbate liver damage. Liver function monitoring is crucial due to reports of hepatic fibrosis and cirrhosis in long-term users (Yu et al. 2020; Rattanakaemakorn et al. 2021; Rak et al. 2022).

#### Hepatotoxicity

Acitretin is linked to a range of hepatotoxic effects, including transient enzyme elevations and rare cases of severe liver injury. In up to 72% of patients, clinical studies have reported transient increases in ALT, AST, and lactate dehydrogenase (LDH), with the majority of cases resolving upon discontinuation (Sauder et al. 2015; Zhong et al. 2023). However, idiosyncratic reactions, such as mixed hepatocellular-cholestatic injury, have been documented, underscoring the necessity of monitoring both transaminases and cholestatic markers such as alkaline phosphatase (ALP) and gamma-glutamyl transferase (GGT) (Sauder et al. 2015). Severe hepatotoxicity, though rare, includes cases of cirrhosis (Ghavam et al. 2017), fulminant hepatic failure following overdose (Zito et al. 2025), and prolonged liver dysfunction linked to autoimmune cofactors (Rak et al. 2022).

The mechanisms responsible for acitretin-induced hepatotoxicity involve mitochondrial dysfunction, particularly within hepatocytes. Acitretin impairs mitochondrial phosphorylation efficiency, decreases ATP synthesis, and triggers calcium-mediated permeability transition (MPT), resulting in apoptosis and necrosis. These effects are mediated through interactions with the adenine nucleotide translocase (ANT), a key regulator of mitochondrial membrane integrity. Furthermore, acitretin’s ability to reverse-metabolize into etretinate, particularly when combined with alcohol intake, may intensify toxicity by extending systemic exposure. While most hepatic enzyme elevations are transient and reversible, rare cases of progressive injury highlight the role of individual susceptibility, such as genetic predispositions or concurrent autoimmune conditions (Sauder et al. 2015; El-Baba et al. 2022; Rak et al. 2022).

**Fig. 5:**
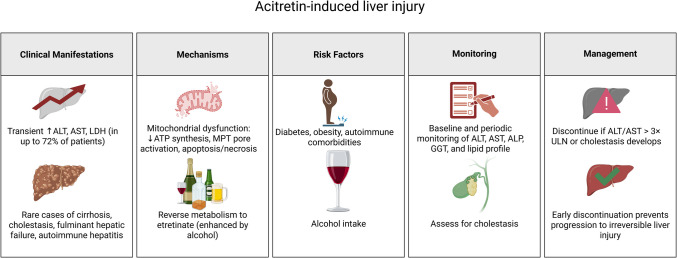
Summary table for acitretin-induced hepatotoxicity. Created in BioRender. Elgindy, A. (2025) https://BioRender.com/20wmrbt

Risk classification and monitoring are essential for reducing hepatotoxicity. Diabetes and obesity are recognized as independent risk factors for hepatic fibrosis in individuals undergoing acitretin and methotrexate treatment; nevertheless, combined therapy does not substantially elevate the risk of fibrosis compared to methotrexate alone (Rattanakaemakorn et al. 2021). Current guidelines recommend baseline and periodic monitoring of liver enzymes, lipid profiles, and cholestatic markers (Sauder et al. 2015; Zhong et al. 2023). While severe hepatotoxicity is uncommon, clinicians must balance acitretin’s therapeutic benefits against its risks, especially in patients with preexisting metabolic conditions. Discontinuation is advised if ALT/AST levels exceed three times the upper limit of normal or if cholestasis develops, as early intervention often prevents progression to irreversible injury (Sauder et al. 2015). A schematic summary of these mechanisms, risk factors, and protective recommendations is illustrated in Fig. 5.

### Apremilast

Apremilast, an oral PDE4 inhibitor, represents a significant advancement in treating moderate-to-severe psoriasis and psoriatic arthritis. Approved by the FDA in 2014, apremilast offers a targeted approach to managing inflammation by modulating intracellular cyclic adenosine monophosphate (cAMP) levels, thereby reducing the production of pro-inflammatory cytokines (Papp et al. 2015; Menter et al. 2020). Unlike traditional systemic therapies, apremilast does not require routine laboratory monitoring for hepatotoxicity or myelosuppression, making it a convenient option for patients with contraindications to methotrexate or biologics (Vangipuram & Alikhan 2017; Li et al. 2018). Its oral administration and favorable safety profile have positioned apremilast as a versatile therapeutic option, particularly for patients with comorbid conditions such as hepatic impairment or metabolic syndrome (Gisondi & Girolomoni 2016).

#### Mechanism of action

Apremilast, an oral small-molecule PDE4 inhibitor, exerts its therapeutic effects by modulating intracellular cAMP concentrations. Inhibition of PDE4 impairs cAMP degradation, resulting in increased intracellular cAMP levels, which subsequently activate protein kinase A (PKA) and attenuate pro-inflammatory signaling pathways, such as NF-κB and mitogen-activated protein kinases (MAPKs). This suppression reduces the production of key cytokines implicated in psoriasis, such as TNF-α, IL-17, IL-23, and IFN-γ, thereby attenuating keratinocyte hyperproliferation and immune cell infiltration (Li et al. 2018; Vujic et al. 2018; Tsentemeidou et al. 2022).

Apremilast targets the IL-23/IL-17 axis, a pivotal pathway in psoriatic inflammation. Apremilast disrupts the feedback loop sustaining psoriatic plaques by inhibiting dendritic cell activation and Th17 differentiation (Schon & Erpenbeck 2018; Ben Abdallah et al. 2021; Honma & Hayashi 2021). Previous studies have demonstrated its ability to downregulate psoriasis-associated genes (e.g., S100A7, DEFB4) (Schafer et al. 2019; Sobolev et al. 2022) and reduce epidermal thickness, correlating with improved PASI scores (LeQuang 2017).

#### Efficacy

Apremilast, an oral PDE4 inhibitor, has shown considerable effectiveness in managing moderate-to-severe plaque psoriasis. Clinical trials and real-world studies have consistently demonstrated enhancements in PASI scores, alongside a favorable safety profile relative to conventional systemic therapies. A long-term real-world study showed that apremilast maintains efficacy for up to 52 weeks, exhibiting sustained PASI reductions and favorable tolerability (Radi et al. 2021). A large-scale phase III randomized trial also reported significant PASI 75 responses in patients treated with apremilast, with the best outcomes seen at the 30 mg twice-daily dose (Paul et al. 2015).

Investigations have confirmed apremilast’s clinical benefits, particularly for patients requiring systemic therapy with a lower risk of immunosuppression. A prospective study evaluating apremilast in psoriasis patients reported sustained PASI75 responses in more than 60% of patients after 40 weeks of therapy (Vujic et al. 2018). A phase 2b randomized controlled trial demonstrated the efficacy of apremilast, with PASI-75 response rates of 23.5% for the 20 mg dose and 28.2% for the 30 mg dose at 16 weeks. These responses were sustained through week 68, highlighting the durability of apremilast’s therapeutic effects over an extended period. Additionally, apremilast exhibited good tolerability, with a favorable safety profile characterized by mild-to-moderate adverse events, such as gastrointestinal symptoms, which were generally transient and manageable (Ohtsuki et al. 2017). Another study highlighted apremilast’s strong drug survival rates, with 75% of patients continuing treatment beyond 52 weeks, attributed to its stable efficacy and favorable tolerability. By week 16, nearly 60% of patients achieved a ΔPASI75 response, underscoring apremilast’s ability to deliver significant and sustained improvements in psoriasis severity while maintaining a well-tolerated safety profile (Papadavid et al. 2018). These findings highlight apremilast as an effective long-term option for psoriasis management, particularly for patients who cannot tolerate biologics or require an oral alternative.

Apremilast has been studied in combination with other therapies to enhance clinical efficacy in plaque psoriasis. It can be safely combined with phototherapy, systemic agents, or biologics, particularly in patients with inadequate responses to monotherapy. Gastrointestinal side effects, though common, are generally manageable (AbuHilal et al. 2016). Apremilast also shows promise as maintenance therapy, preventing flare-ups after cyclosporine withdrawal and delaying the need for biologics. It is effective for patients who are unresponsive to biologics and can be combined with biologics or systemic therapies (e.g., methotrexate), offering synergy and a better safety profile. Recent evidence supports its use in recalcitrant psoriasis, enabling dose reduction of conventional agents to minimize side effects (Torres & Puig 2018). Additionally, apremilast’s efficacy extends beyond plaque psoriasis to difficult-to-treat cases such as palmoplantar and scalp psoriasis, where significant symptom improvement has been documented (Rich et al. 2016; Keating 2017).

### Hepatotoxicity

Apremilast is generally recognized for its favorable hepatic safety profile, particularly compared to systemic therapies like methotrexate and acitretin. Unlike traditional immunosuppressants, apremilast does not require routine liver function monitoring, as clinical studies have not shown a significant risk of hepatotoxicity. However, some real-world data and case reports indicate minimal changes to liver function tests in a subset of patients, emphasizing the need for careful evaluation in individuals with pre-existing hepatic conditions (Vangipuram & Alikhan 2017; Li et al. 2018; Balak et al. 2021; Jadhav et al. 2024).

A comparative evaluation of liver function tests in psoriasis patients treated with apremilast and tofacitinib found that apremilast users occasionally experienced mild, transient elevations in ALT and AST levels, which rarely required discontinuation. In contrast, tofacitinib was associated with more significant elevations in liver function tests, highlighting apremilast’s relatively favorable hepatic safety profile (Jadhav et al. 2024). Another study analyzing the hepatic safety of systemic psoriasis treatments reported no significant hepatotoxic effects with apremilast compared to methotrexate and acitretin, which are well-documented for their potential liver toxicity (Munera-Campos et al. 2022).

Apremilast has been effectively utilized in special populations, including liver transplant recipients, without causing adverse hepatic effects. A case report demonstrated its efficacy in controlling psoriasis flares following transplantation, underscoring its safety in individuals with significant hepatic impairment (Rosi et al. 2023). Additionally, an Indian expert panel emphasized that apremilast is not contraindicated in patients with liver disease and can be safely administered without dose adjustments in patients with hepatic impairment (Rajagopalan et al. 2021). While significant studies affirm its hepatic safety, additional long-term research is essential to comprehensively assess its effects on liver health, particularly in individuals with predisposing risk factors.

## Balancing efficacy and hepatotoxicity

The selection of an oral medication for psoriasis treatment requires a careful balance between therapeutic efficacy and the risk of hepatotoxicity. Table 3 summarizes the efficacy and hepatotoxicity risks of commonly used oral systemic treatments, highlighting key clinical outcomes and safety considerations.

**Table 3:** Comparison of oral psoriasis treatments: efficacy and hepatotoxicity risks

Medication	Mechanism of action	PASI 75 response rate	Onset of action	Hepatotoxicity risk	Key considerations	References
Methotrexate	Folate antagonist inhibiting DNA synthesis and immune response	~ 40–50% in 16 weeks	8–12 weeks	High—risk of hepatic fibrosis and cirrhosis	Requires liver function monitoring; contraindicated in patients with existing liver disease	Dogra et al. (2021); Ezhilarasan (2021); Schmidt et al. (2022); Abdallah et al. (2024); Sweilam et al. (2024)
Acitretin	Binding to RARs and RXRs to normalize keratinocyte differentiation	~ 40–60% in 12–16 weeks	4–8 weeks	Moderate-high—associated with elevated liver enzymes and rare cases of hepatic fibrosis	Avoid alcohol to prevent conversion to etretinate; monitor liver enzymes	Sauder et al. (2015); Yu et al. (2020); Rattanakaemakorn et al. (2021); Rak et al. (2022); Zhong et al. (2023)
Cyclosporine	Calcineurin inhibitor, suppressing T-cell activation	~ 60–80% in 12 weeks	4–6 weeks	Moderate—liver function abnormalities reported, but less frequent than with methotrexate	Short-term use is recommended to minimize toxicity; monitor renal function	Akool el (2015); Korolczuk et al. (2016); Singh and Argáez (2018); Balak et al. (2020); El-Mancy et al. (2022); S. A. Faheem et al. (2022a, b); Dogra et al. (2024)
Apremilast	PDE4 inhibitor reduces pro-inflammatory cytokine production	~ 30–40% in 16 weeks	2–4 weeks	Low—minimal hepatotoxicity risk; occasional transient liver enzyme elevations	No routine liver function monitoring required; suitable for patients with liver disease	Papp et al. (2015); Rich et al. (2016); Vangipuram and Alikhan (2017); Li et al. (2018); Reich et al. (2018); Afra et al. (2019); Balak et al. (2021); Rajagopalan et al. (2021); Munera-Campos et al. (2022); Rosi et al. (2023); Jadhav et al. (2024)

### Comparison of efficacy among oral medications

The efficacy of oral systemic medications for psoriasis varies depending on their mechanism of action, patient response, and disease severity. Methotrexate remains a cornerstone in psoriasis management, achieving PASI 75 in about 40–50% of patients after 16 weeks of therapy. However, its long-term use is associated with hepatotoxicity and cumulative dose-related liver fibrosis, necessitating careful monitoring. (Kaushik & Lebwohl 2019b; Dogra et al. 2022). In contrast, acitretin, a systemic retinoid, is particularly effective in treating pustular and erythrodermic psoriasis, with PASI 75 response rates of 40–60% in 12–16 weeks. Acitretin’s efficacy is often enhanced when combined with phototherapy, but its side effect profile, including mucocutaneous dryness and hepatotoxicity, limits its widespread use as monotherapy (Sauder et al. 2015; Pai et al. 2019; Nakamura & Koo 2020; Arora et al. 2021; Rattanakaemakorn et al. 2021).

Cyclosporine demonstrates rapid and high efficacy, with PASI 75 rates of 60–80% at 12 weeks. However, its use is limited to short-term therapy due to nephrotoxicity and hypertension risks (Singh & Argáez 2018; Balak et al. 2020; Rajagopalan et al. 2022; Ruggiero et al. 2023; Dogra et al. 2024). In comparison, apremilast offers a safer long-term alternative with PASI 75 response rates of 30–40% at 16 weeks. Although its efficacy is lower than methotrexate and cyclosporine, apremilast’s favorable safety profile, particularly its low hepatotoxicity risk, makes it a valuable option for patients who require long-term systemic therapy and cannot tolerate immunosuppressants (Papp et al. 2015; Rich et al. 2016; Vangipuram & Alikhan 2017; Li et al. 2018; Reich et al. 2018; Afra et al. 2019; Balak et al. 2021; Jadhav et al. 2024).

### Comparison of hepatotoxicity risks

Hepatotoxicity is a significant concern when selecting oral systemic therapies for psoriasis, as some medications pose a substantial risk of liver damage, necessitating regular monitoring. Among oral treatments, methotrexate is the most strongly associated with hepatotoxicity, with long-term use linked to hepatic fibrosis and cirrhosis. The risk increases with alcohol consumption, type 2 diabetes, and obesity, leading to the recommendation of regular liver function tests and imaging for long-term users (Ezhilarasan 2021; Koutsompina et al. 2021; Schmidt et al. 2022; Atallah et al. 2023; Sweilam et al. 2024; Ali et al. 2025).

Acitretin carries a significant risk of hepatotoxicity, with up to one-third of patients treated with acitretin experiencing elevated liver enzymes. Rare cases of hepatic fibrosis have been reported, particularly in patients consuming alcohol, which enhances the drug’s conversion to etretinate, a more hepatotoxic metabolite. Regular liver function tests monitoring is recommended, especially during long-term therapy (Sauder et al. 2015; Ghavam et al. 2017; Yu et al. 2020; Lauer et al. 2021; Rattanakaemakorn et al. 2021).

Cyclosporine, though primarily nephrotoxic, has been associated with mild hepatic dysfunction, including transient bilirubin and transaminase elevations. However, these effects are generally reversible upon dose reduction (Singh & Argáez 2018; Wu et al. 2018; Nsengimana et al. 2022; Rajagopalan et al. 2022; Lv et al. 2024).

In contrast, apremilast is considered the safest option regarding hepatotoxicity. Unlike methotrexate and acitretin, apremilast does not require routine liver function monitoring, as studies have shown no significant liver toxicity in long-term users. A comparative study reported minimal liver enzyme elevations, with no cases of severe hepatic injury (Mayba & Gooderham 2017; Kavanaugh et al. 2019; Jadhav et al. 2024). Additionally, apremilast has been successfully used in psoriasis patients with pre-existing liver disease, reinforcing its hepatic safety profile (Palmou-Fontana et al. 2019; Rosi et al. 2023).

Given the varying levels of hepatotoxic risk among these oral medications, individualized patient assessment is essential when selecting treatment options. Factors such as pre-existing liver conditions, alcohol consumption, obesity, diabetes, and concomitant use of other hepatotoxic drugs should be carefully evaluated.

### Risk mitigation strategies

#### Monitoring and drug-specific risk considerations

Minimizing hepatotoxicity risks in psoriasis treatment requires a comprehensive approach that includes regular monitoring, hepatoprotective agents, lifestyle modifications, and dose adjustments. Among oral systemic therapies, methotrexate and acitretin pose the highest hepatotoxic risks, necessitating frequent liver function tests and preventive measures. For methotrexate, long-term therapy demands fibrosis risk assessment through imaging or non-invasive biomarkers, alongside folic acid supplementation to reduce toxicity (Bafna et al. 2021; Clary et al. 2021; Rattanakaemakorn et al. 2021).

Non-invasive methods for assessing liver fibrosis are increasingly recommended in patients receiving systemic therapies with known hepatotoxic potential. Among these, transient elastography (TE) has demonstrated high reliability in detecting methotrexate-induced hepatic fibrosis and is often preferred over liver biopsy when combined with other markers. In a study of psoriasis patients, TE detected liver fibrosis in 36.1% of those treated with methotrexate, compared to 19.6% in methotrexate-naïve controls. The study also evaluated the aspartate aminotransferase-to-platelet ratio index (APRI) and the Fibrosis-4 (FIB-4) index, finding that TE had a higher detection rate for fibrosis than these serum-based markers (Lee et al. 2022).

Acitretin, on the other hand, carries an increased risk of hepatotoxicity when combined with alcohol, as it converts into etretinate, the more toxic and persistent metabolite. This highlights the importance of patient education on alcohol avoidance and adherence to monitoring protocols to ensure safe and effective treatment outcomes (Sauder et al. 2015; Szentkereszty-Kovacs et al. 2021; Zito et al. 2025).

Cyclosporine is highly effective for rapid symptom control in psoriasis and poses a lower risk of hepatotoxicity compared to methotrexate (Das et al. 2023). However, it requires careful monitoring due to its potential for nephrotoxicity and hypertension (Rajagopalan et al. 2022). Liver function should also be assessed, especially in high-risk patients, such as those with obesity (Fiore et al. 2018; Nast et al. 2020). Regular evaluations of serum creatinine and blood pressure are crucial, with dose adjustments made as needed to minimize adverse effects (Fiore et al. 2018; Hong et al. 2019; Nast et al. 2020). Cyclosporine is generally reserved for short-term use or as a bridge therapy, particularly in patients without pre-existing renal or cardiovascular conditions, to ensure its benefits outweigh the risks.

#### Emerging hepatoprotective agents

Hepatoprotective agents have been explored to counteract drug-induced liver injury from psoriasis drugs. Silymarin, a potent antioxidant, has demonstrated hepatoprotective and nephroprotective properties by reducing oxidative stress and fibrosis in methotrexate-induced liver and renal injury (Dabak & Kocaman 2015; Khokhar et al. 2017; Ahmed et al. 2023). Similarly, N-acetylcysteine, a GSH precursor, has been studied for its ability to ameliorate oxidative liver damage (Demirci et al. 2019; Eroglu et al. 2020). Glycyrrhizin, a compound derived from licorice root, has been reported to reduce transaminase levels in acitretin-treated psoriasis patients, suggesting a protective role against retinoid-induced hepatotoxicity (Yu et al. 2020).

Several hepatoprotective agents, including natural products and dietary interventions, are increasingly gaining attention. Curcumin, in particular, has been widely recognized for its anti-inflammatory and hepatoprotective properties. Clinical and preclinical studies have demonstrated its potential to reduce elevated liver enzyme levels associated with hepatotoxic medications (Farkhondeh & Samarghandian 2016; A Hussein et al. 2018; Jarhahzadeh et al. 2021; Hasan Khudhair et al. 2022). Similarly, resveratrol has demonstrated liver-protective benefits by modulating oxidative stress and inflammatory pathways (Faghihzadeh et al. 2015; Bingul et al. 2021; Özgöçmen & Yeşilot 2021).

Several additional hepatoprotective agents have shown promise in reducing methotrexate-induced liver injury, including melatonin (Abdallah et al. 2024), punicalagin (Al-Khawalde et al. 2022), glabridin (Dogra et al. 2021), naringin (Elsawy et al. 2020), vitamin C (Hasan Khudhair et al. 2022), ferulic acid (Roghani et al. 2020), pine resin (Sweilam et al. 2024), and magnesium isoglycyrrhizinate (Xia et al. 2022). Similarly, for cyclosporine-induced hepatotoxicity, compounds such as wheat germ oil (Akool el 2015), avocado seeds (El-Magd et al. 2022), alpha-lipoic acid (El-Mancy et al. 2022), vildagliptin (El-Sherbeeny & Nader 2016), and pyrvinium pamoate (S. A. Faheem et al. 2022a, b) have exhibited protective effects by reducing oxidative stress, apoptosis, and inflammation. Despite the promising preclinical data, further large-scale clinical trials are warranted to establish the efficacy and safety of these agents in psoriasis patients receiving systemic therapies.

#### Lifestyle modifications

Lifestyle modifications also play a critical role in reducing hepatotoxic risks. Patients on methotrexate and acitretin should be advised to avoid alcohol and maintain a healthy weight, as NAFLD is a common comorbidity in psoriasis patients, exacerbating liver dysfunction (Balak et al. 2021; Rattanakaemakorn et al. 2021; Di Martino et al. 2023). Regular physical activity and a Mediterranean-style diet rich in antioxidants may further reduce liver inflammation and enhance overall hepatic health while supporting the therapeutic efficacy of treatment (Abenavoli et al. 2017; Katsimbri et al. 2021).

### Patient management and monitoring

Effective management and monitoring of patients receiving oral treatments for psoriasis are crucial for minimizing adverse effects, particularly hepatotoxicity, and maximizing therapeutic benefits. Before systemic therapy is initiated, it is essential to perform comprehensive baseline assessments, which include liver function tests, complete blood counts, and renal function tests. Patients with risk factors such as obesity, metabolic syndrome, excessive alcohol consumption, or preexisting liver disease require additional monitoring, as they are more susceptible to drug-induced liver injury (Fiore et al. 2018; Nast et al. 2020). Methotrexate, which carries the highest hepatotoxic risk among oral treatments, necessitates routine liver function testing at least every 3 months, while cyclosporine requires close monitoring of renal function and blood pressure due to its nephrotoxic potential (Warren et al. 2016; Nast et al. 2020). For acitretin, periodic liver function tests and lipid profiles are crucial, as the drug is associated with hyperlipidemia and hepatotoxicity (Nast et al. 2020; Balak et al. 2021), while patients on apremilast require assessment for gastrointestinal side effects; they do not need routine liver monitoring (Rosi et al. 2023).

In cases of drug-induced hepatotoxicity, dose adjustments, treatment discontinuation, or alternative therapies may be necessary. Methotrexate-induced liver enzyme elevations often resolve with temporary withdrawal or folic acid supplementation, which mitigates hepatic toxicity without compromising efficacy (Balak et al. 2020). Cyclosporine-induced liver dysfunction, although less common, can be managed by dose reduction or switching to alternative medications such as adalimumab, while antioxidant supplementation such as vitamin E and N-acetylcysteine has shown potential in mitigating oxidative liver damage (Ighani et al. 2019; Safaa Ahmed Faheem et al. 2022a, b). Acitretin users must strictly avoid alcohol, as it enhances the drug’s conversion to etretinate, a more hepatotoxic metabolite, leading to prolonged systemic exposure and liver toxicity (Rak et al. 2022; van de Kerkhof 2023). Apremilast, with its favorable safety profile, is a preferred option for patients with preexisting liver disease or those who experience hepatotoxicity from other systemic agents (Rajagopalan et al. 2021; Munera-Campos et al. 2022; Rosi et al. 2023; Jadhav et al. 2024).

Patient education is crucial for treatment success, emphasizing adherence to prescribed regimens, early recognition of liver dysfunction, and necessary lifestyle modifications. A balanced diet, alcohol restriction, and regular exercise can help mitigate the risk of hepatotoxicity, particularly in patients with NAFLD, a common comorbidity in psoriasis. Clinicians should adopt shared decision-making strategies to ensure patients are well-informed about potential side effects and the importance of regular follow-ups. This proactive approach facilitates early detection of adverse effects, timely therapeutic adjustments, and improved long-term outcomes in psoriasis management.

## Emerging therapies and future directions in psoriasis management

### Advancements in targeted and personalized psoriasis therapies

Novel pathways and personalized approaches are the primary focus of emerging therapies for psoriasis, to enhance efficacy and minimize adverse effects. Targeting the IL-23/Th17 axis, crucial for developing psoriasis, is one of the most promising research areas. IL-23 inhibitors, including mirikizumab, have demonstrated impressive efficacy in clinical trials, resulting in substantial improvements in psoriatic skin lesions (Blauvelt et al. 2022; Masa’deh 2024). These agents target upstream cytokines, offering longer dosing intervals and sustained remission even after discontinuation (K. Yang et al. 2021a, b). Additionally, small-molecule inhibitors, such as retinoic acid receptor-related orphan receptor gamma-t (RORγt) inhibitors (e.g., VTP-43742) and tyrosine kinase 2 (TYK2) inhibitors (e.g., deucravacitinib), are currently in development. These inhibitors have the potential to exert immunomodulatory effects on cytokine signaling pathways and cell proliferation (Pandya et al. 2018; Krueger et al. 2022; Strober et al. 2023). These advancements underscore the transition to more precise and efficient therapies, which enhance patient outcomes and reduce systemic inflammation.

The development of new biologic agents and small-molecule inhibitors is revolutionizing the treatment of psoriasis. IL-17 inhibitors, including brodalumab, ixekizumab, and secukinumab, have exhibited sustained efficacy and a rapid onset of action, particularly in the treatment of nail psoriasis (Lopez-Ferrer et al. 2015; Reich et al. 2017; Foulkes & Warren 2019). However, the long-term safety profiles of comorbidities such as IBD and challenges such as mucocutaneous candidiasis are critical concerns that require further investigation (Davila-Seijo et al. 2017; Reich et al. 2017; Foulkes & Warren 2019). Another promising class of agents includes IL-36 inhibitors, which target the IL-36 signaling pathway implicated in generalized pustular psoriasis (GPP). Spesolimab, a monoclonal antibody against IL-36R, has shown efficacy in early-phase trials, with ongoing phase II and III studies evaluating its potential in GPP (Thakur & Mahajan 2022; Ali et al. 2023; Bukhari et al. 2024). These new agents, along with Rho-associated kinase (ROCK)2 inhibitors like KD025, which modulate inflammatory responses by targeting Th17 cells, represent a significant leap forward in psoriasis therapeutics (Zanin-Zhorov et al. 2017).

Personalized medicine is becoming increasingly crucial in psoriasis management, focusing on tailoring treatments based on individual genetic, immunological, and clinical profiles. Biomarkers such as HLA-C*06:02, IL-23R, and IL-17A are being explored to predict treatment response and disease severity (Pourani et al. 2022; Masa’deh 2024). Multi-omics technologies, including genomics, transcriptomics, and proteomics, are providing deeper insights into the molecular mechanisms of psoriasis, facilitating the development of targeted therapies (Pourani et al. 2022; Ramchandani & Goyal 2024; Rusinol & Puig 2024). For instance, identifying loss-of-function mutations in the *IL-36Ra* gene has led to developing IL-36 inhibitors for patients with GPP (Johnston et al. 2017; Todorovic et al. 2019; Sugiura 2022). Furthermore, advancements in topical treatments, such as microneedles and nanoparticle-based drug delivery systems, are enhancing the precision and efficacy of localized therapies, particularly for difficult-to-treat areas like the scalp and nails (Chiu et al. 2015; Lee & Prausnitz 2018; Shravanth et al. 2021; D. Yang et al. 2021a, b). A summary of novel therapeutic approaches, including biologics, small-molecule inhibitors, and personalized treatment strategies, is provided in Table 4.

**Table 4:** Emerging and targeted therapies in psoriasis

Category	Agents	Target	Key findings/advantages	References
IL-23 inhibitors	Mirikizumab	IL-23/Th17 axis inhibition	High efficacy, long dosing intervals, sustained remission post-discontinuation	K. Yang et al. (2021a, b); Blauvelt et al. (2022); Masa’deh (2024)
Small-molecule inhibitors	RORγt inhibitors (VTP-43742), TYK2 inhibitors (deucravacitinib)	RORγt/TYK2 inhibition	Immunomodulatory effects on cytokine signaling pathways	Pandya et al. (2018); Krueger et al. (2022); Strober et al. (2023)
IL-17 inhibitors	Brodalumab, ixekizumab, secukinumab	IL-17 blockade	Rapid onset, sustained efficacy (especially for nail psoriasis)	Lopez-Ferrer et al. (2015); Reich et al. (2017); Foulkes and Warren, (2019)
IL-36 inhibitors	Spesolimab (anti-IL-36R)	IL-36 pathway inhibition	Effective in generalized pustular psoriasis (GPP)	Thakur and Mahajan (2022); Ali et al. (2023); Bukhari et al. (2024)
ROCK2 inhibitors	KD025	Th17 cell modulation	Reduces inflammatory responses	Zanin-Zhorov et al. (2017)
Personalized medicine	Biomarkers (HLA-C*06:02, IL-23R, IL-17A)	Genetic/immunological profiling	Predicts treatment response and severity	Pourani et al. (2022); Masa’deh (2024)
Topical innovations	Microneedles, nanoparticle-based delivery	Enhanced drug penetration	Improves treatment for scalp/nail psoriasis	Chiu et al. (2015); Lee and Prausnitz (2018); Shravanth et al. (2021); D. Yang et al. (2021a, b)

### Hepatic safety profiles of emerging systemic therapies

Deucravacitinib has demonstrated a favorable hepatic safety profile across multiple clinical trials, with ALT and AST elevations being generally mild, transient, and often related to underlying liver conditions or concomitant medications (Lebwohl et al. 2023; Zhang et al. 2025). Over periods ranging from 1 to 4 years, no significant hepatotoxicity was observed, and incidences of ALT and AST elevations greater than three times the upper limit of normal (ULN) were rare. Most abnormal liver function events were mild, and no cases met the criteria for potential drug-induced liver injury/Hy’s law. Long-term data suggest that routine laboratory monitoring may not be necessary during deucravacitinib treatment (Korman et al. 2024; Strober et al. 2024; Armstrong et al. 2025).

Secukinumab has demonstrated a favorable hepatic safety profile, with no significant hepatotoxicity reported in clinical trials. In a controlled, open trial with psoriasis patients, secukinumab did not cause liver enzyme elevations, while methotrexate led to a threefold increase in liver enzymes in 4 of 64 patients, necessitating withdrawal (Gisondi et al. 2020b, c, a). In combination with tretinoin, secukinumab effectively reduced inflammation without affecting liver function or metabolism (Chen et al. 2024). In patients with HBV infection, secukinumab showed good efficacy, but periodic monitoring of liver function is recommended as reactivation of HBV has been observed, particularly in susceptible individuals, with a reported reactivation rate of 15.2% in psoriasis patients (Chiu et al. 2018; Qin et al. 2022).

Ixekizumab has demonstrated a low incidence of hepatic events, with a 12-week observational study reporting mild hepatic events in 0.9% of Chinese adults with moderate-to-severe plaque psoriasis, none meeting the criteria for severe hepatotoxicity (Y. Li et al. 2024a, b). Brodalumab has not been associated with clinically significant liver injury in clinical trials; however, the potential link between brodalumab and autoimmune liver diseases, such as AIH/PBC overlap syndrome, requires further investigation, and caution is advised in patients at risk (Girard 2019; Okazaki et al. 2021). Spesolimab was evaluated in a phase I study involving healthy Chinese subjects. The study reported that spesolimab was well tolerated, with no significant hepatic adverse events observed (Cao et al. 2024).

### Pharmacogenomic predictors of hepatotoxicity

Recent pharmacogenomic research has identified several genetic polymorphisms that influence both the efficacy and hepatotoxicity of methotrexate in psoriasis treatment. Notably, the MTHFR rs1801133 TT genotype has been associated with higher PASI 90 response rates, indicating better therapeutic outcomes, while the CT + TT genotypes correlate with an increased risk of liver function abnormalities in patients with psoriatic arthritis (Zhu et al. 2022). Similarly, the MTHFR rs1801131 CT genotype has been linked to lower PASI 75 response rates and a reduced risk of ALT elevation, suggesting a complex relationship between MTHFR polymorphisms and both efficacy and hepatotoxicity (Wang et al. 2023). Beyond MTHFR, polymorphisms in genes encoding drug transporters, such as SLC19A1 (rs1051266) and SLCO1B1 (SLCO1B15 and SLCO1B115), have been implicated in MTX transport efficiency and toxicity profiles. Variants in SLC19A1 have been associated with impaired MTX transport and resistance, while SLCO1B1 low-activity haplotypes have been linked to delayed MTX clearance and increased toxicity risk (Wang et al. 2023).

## Future perspectives

As the therapeutic landscape for psoriasis continues to evolve, future efforts should prioritize the development of safer oral systemic agents with reduced hepatotoxicity profiles, particularly for use in long-term management. Research should focus on elucidating genetic and metabolic predictors of drug-induced liver injury, enabling early identification of high-risk individuals. The integration of pharmacogenomic profiling holds promise for tailoring therapy based on individual susceptibility to hepatotoxic effects. In parallel, advancements in non-invasive hepatic monitoring tools, such as elastography and serum fibrosis indices, should be further validated and incorporated into routine clinical practice. Additionally, combination regimens that optimize efficacy while minimizing cumulative hepatic burden and the evaluation of hepatoprotective adjuvants warrant further investigation. These strategies will support a shift toward precision medicine approaches, ultimately improving treatment safety and patient quality of life.

## Conclusion

Psoriasis is a chronic inflammatory condition that demands long-term therapeutic strategies, with oral systemic drugs remaining central to its management. This review has highlighted the importance of clinical efficacy and hepatic safety across commonly used agents, including methotrexate, cyclosporine, acitretin, and apremilast. While effective in controlling disease activity, agents such as methotrexate and acitretin pose significant hepatotoxic risks. This underscores the need for routine monitoring and careful patient selection, particularly in those with metabolic or hepatic comorbidities. Cyclosporine, though potent, requires vigilance for nephrotoxicity and hypertension, whereas apremilast offers a safer hepatic profile.

Emerging therapies, including TYK2 and RORγt inhibitors, offer new hope with potentially improved safety margins, yet their long-term hepatic effects remain to be fully elucidated. Recent mechanistic insights have expanded our understanding of hepatotoxicity, facilitating the development of biomarkers, protective strategies, and non-invasive diagnostics. Ultimately, optimal psoriasis management should adopt a multidisciplinary, individualized approach that balances therapeutic benefit with hepatotoxic risk. Leveraging precision medicine, pharmacogenomics, and validated monitoring tools will be essential in minimizing liver injury and enhancing outcomes in systemic psoriasis care.

## Data Availability

All source data for this work (or generated in this study) are available upon reasonable request.
